# Theoretical Study on ORR/OER Bifunctional Catalytic Activity of Axial Functionalized Iron Polyphthalocyanine

**DOI:** 10.3390/molecules29010210

**Published:** 2023-12-30

**Authors:** Guilin Wang, Xiaoqin Feng, Rongrong Ren, Yuxin Wang, Jie Meng, Jianfeng Jia

**Affiliations:** 1 Key Laboratory of Magnetic Molecules and Magnetic Information Materials (Ministry of Education), School of Chemistry and Material Science, Shanxi Normal University, Taiyuan 030031, China; wangguilin@ycu.edu.cn (G.W.); 17636260644@163.com (X.F.); 18435202416@163.com (R.R.); wangyux820@163.com (Y.W.); 17731003064@163.com (J.M.); 2 Department of Physics and Electronic Engineering, Yuncheng University, Yuncheng 044000, China

**Keywords:** axial ligands, ORR/OER bifunctional catalytic activity, density functional theory

## Abstract

Designing efficient ORR/OER bifunctional electrocatalysts is very significant for reducing energy consumption and environmental protection. Hence, we studied the ORR/OER bifunctional catalytic activity of iron polyphthalocyanine (FePPc) coordinated by a series of axial ligands which has different electronegative coordination atom (FePPc-L) (L = -CN, -SH, -SCH_3_, -SC_2_H_5_, -I, -Br, -NH_2_, -Cl, -OCH_3_, -OH, and -F) in alkaline medium by DFT calculations. Among all FePPc-L, FePPc-CN, FePPc-SH, FePPc-SCH_3_, and FePPc-SC_2_H_5_ exhibit excellent ORR/OER bifunctional catalytic activities. Their ORR/OER overpotential is 0.256 V/0.234 V, 0.278 V/0.256 V, 0.280 V/0.329 V, and 0.290 V/0.316 V, respectively, which are much lower than that of the FePPc (0.483 V/0.834 V). The analysis of the electronic structure of the above catalysts shows that the electronegativity of the coordination atoms in the axial ligand is small, resulting in less distribution of dz^2^, dyz, and dxz orbitals near E_f_, weak orbital polarization, small charge and magnetic moment of the central Fe atom, and weak adsorption strength for *OH. All these prove that the introduction of axial ligands with appropriate electronegativity coordinating atoms can adjust the adsorption of catalyst to intermediates and modify the ORR/OER bifunctional catalytic activities. This is an effective strategy for designing efficient ORR/OER bifunctional electrocatalysts.

## 1. Introduction

Due to the increase in fossil fuel emissions, the energy crisis and global environmental pollution problems have become increasingly prominent. At present, fuel cells are considered an effective substitute for fossil fuels, because they can protect natural resources and the environment [1,2,3]. Oxygen reduction reaction (ORR) [4] and oxygen evolution reaction (OER) [5] are two important reactions in fuel cells. Up to now, it has been proven that Pt-based precious metals are the most efficient electrocatalysts for ORR [6,7]. IrO_2_ and RuO_2_ are classic OER electrocatalysts [8,9]. However, their high cost and low reserves hinder wide application. Thus, it is necessary to explore other non-precious catalysts to replace precious metal catalysts. People have been studying all kinds of non-precious catalysts of ORR and OER [10,11,12,13,14]. Transition metal phthalocyanine (TMPc)-based electrocatalysts have been of broad concern due to their low preparation cost, high conjugated structure, high thermal stability, chemical stability, and excellent catalytic performance [15]. Since 1964, cobalt phthalocyanine molecule was used as an ORR catalyst [16], TMPcs have been extensively studied as superior ORR [17,18,19], OER [20,21,22], and ORR/OER [23,24,25,26] difunctional catalysts. Transition metal polyphthalocynine complexes (TMPPc) formed by multiple TMPcs show better conductivity, stability, and electrocatalytic activity than that of TMPc monomers. Previous research has proved that TMPPc can be used as promising ORR [27,28,29,30], OER [31,32], and ORR/OER difunctional electrocatalysts [33]. For instance, a conjugated aromatic network two-dimensional material composed of TMPPc (TM = Fe, Co, Fe/Co) with a large-conjugated plane, highly exposed active sites, and strong conductivity exhibited outstanding ORR performance [27]. It was reported that a thinner edge-anhydride-functionalized CoPPc with more exposed active sites and wider interlayer spacing showed better ORR catalytic performance [28]. Meanwhile, polymeric azo linkage cobalt phthalocyanine was synthesized by the diazotization method. It was mixed with the benchmark catalyst IrO_2_ and showed a good OER catalytic performance [31]. In addition, it was reported a battery of polymeric cobalt phthalocyanines containing S or SO_2_ linkers coated on various substrates showed good OER activity [32]. Especially, FePPc has been recognized as a promising ORR electrocatalyst both experimentally and theoretically [27,29,34]. However, FePPc is not a good OER electrocatalyst on account of the high OER overpotential [33]. It is always the goal of people to explore or design a low-cost and efficient ORR/OER bifunctional electrocatalyst. Therefore, it is very meaningful to find an efficient strategy to make FePPc become an excellent ORR/OER bifunctional electrocatalyst.

Many studies have reported that introducing axial ligands to the active site is a very useful way to improve the electrocatalytic activity of MN_4_ complexes. The axial ligands can change the electron density of the central metal to influence the binding energy between the central metal atom and the reaction intermediate [35,36]. For example, a biomimetic iron phthalocyanine with pyridine functionalized carbon nanotubes as an axial ligand on the Fe atom was synthesized and exhibited better ORR catalytic activity compared with Pt/C catalyst. The higher ORR activity was mainly due to the adjustment of the electronic structure of the Fe atom by axial ligands [37]. Three different penta-coordinated FePc electrocatalysts were successfully prepared and showed better ORR catalytic performance than the FePc [38]. The influence of axial ligands on the ORR catalytic performance of FePPc was studied by introducing a series of axial ligands (L = -F, -Cl, -Br, -I, -OH et al.) to Fe atom of FePPc to form FePPc-L in the experiment. It was found that FePPc-I showed good stability and ORR catalytic performance [35]. It was also proved that appropriate axial ligands can promote the ORR catalytic activity of FeN_4_ [36].

Thus, it can be seen that the introduction of axial ligands with different electronegative coordination atoms on Fe atoms in FePPc can affect the electronic structure of the FeN_4_ active center of FePPc in different degrees, thereby affecting the catalytic activity of FePPc. Therefore, in this study, based on DFT calculations, we introduced a series of axial ligands (L = -CN, -SH, -SCH_3_, -SC_2_H_5_, -I, -Br, -NH_2_, -Cl, -OCH_3_, -OH, and -F) with different electronegative coordination atoms on the Fe atom of FePPc to form FePPc-L and studied their ORR/OER catalytic activity. It is expected to understand how the electronegativity of coordination atoms in axial ligands affects the catalytic activity of the FePPc and design effective ORR/OER bifunctional catalysts. We look forward to the research that will be instructive to explore the efficient ORR/OER bifunctional electrocatalysts.

## 2. Results and Discussion

### 2.1. Structure and Stability

**Figure 1:**
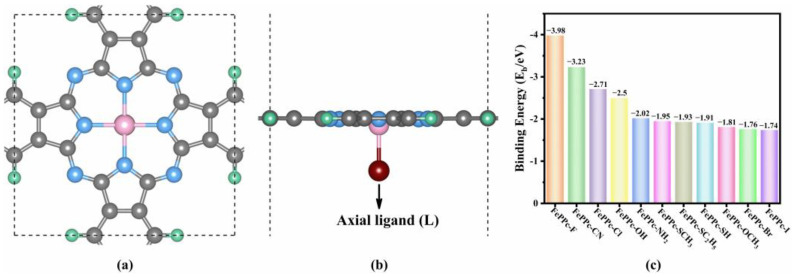
(**a**,**b**) The top and side view of the unit cell of FePPc-L, the green, gray, blue, and pink present H, C, N, and Fe atoms, respectively, and wine red presents the axial ligands. (**c**) Binding energy (E_b_/eV) of FePPc-L.

The top and side view of the optimized geometrical configuration of the unit cells of FePPc-L (L = -CN, -SH, -SCH_3_, -SC_2_H_5_, -I, -Br, -NH_2_, -Cl, -OCH_3_, -OH, and -F) are shown in Figure 1a,b. In the optimized unit cell of FePPc as shown in Figure S1 in the Supporting Information (SI), the lattice parameters and the average bond length of Fe-N bond are 10.66 Å and 1.93 Å, respectively, which is concordant with prior results [29,34]. The lattice parameters of various FePPc-L are not much different from that of FePPc. However, average bond length of Fe-N (d_Fe-N_) is slightly longer than that of FePPc as shown in Table S1. This is because the interaction between axial ligands and Fe atom weakens the Fe-N bond. The thermodynamic stability of catalysts usually can be assessed by the binding energy (E_b_). The negative E_b_ means that the catalyst is thermodynamically stable, and it can be easily prepared in experiments. The E_b_ of FePPc-L (L = -CN, -SH, -SCH_3_, -SC_2_H_5_, -I, -Br, -NH_2_, -Cl, -OCH_3_, -OH, and -F) are calculated according to the following formula:

E_b_ = E_FePPc-L_ − E_FePPc_ − E_L,_

where E_FePPc-L_, E_FePPc_, and E_L_ are the energy of FePPc-L, FePPc, and isolated axial ligand (L) in the vacuum calculated by DFT calculation. As seen in Figure 1c, the thermodynamic stability of all FePPc-L is good on account of their negative E_b_. Moreover, ORR and OER are performed in an alkaline medium, and there is competition between OH^−^ and axial ligands in the adsorption with the central Fe atom [39]. The E_b_ calculated for FePPc-I is −1.74 eV, which is obviously larger than that of FePPc-OH (−2.50 eV) as seen in Figure 1c. However, FePPc-I was successfully prepared and exhibited higher stability in an alkaline medium and showed higher ORR catalytic activity than that of FePPc-OH [35]. The reason may be that it is not easy for OH^−^ to replace the axial ligand -I due to the comprehensive control of steric hindrance, reaction kinetics, thermodynamic equilibrium, etc. in real experiments, and FePPc-I showed good stability and high ORR catalytic activity in alkaline medium. This can be used as an analogy. Among all FePPc-L considered, FePPc-I had the largest E_b_, other ligands have stronger interaction with Fe atoms than that of FePPc-I. If FePPc-I can stably exist, so may other FePPc-L.

### 2.2. ORR/OER Mechanism of FePPc and FePPc-L

Previous studies [29,35] have proven that the ORR mechanism on FePPc and FePPc-L is the four-electron in an alkaline medium. The OER is the reversible process of the ORR, so we studied the ORR/OER bifunctional catalytic activity of FePPc and FePPc-L (L = -CN, -SH, -SCH_3_, -SC_2_H_5_, -I, -Br, -NH_2_, -Cl, -OCH_3_, -OH, and -F) based on the four-electron mechanism in the alkaline medium in this work.

The ORR in alkaline medium can be described as:

R1: * + O_2_ + H_2_O + e^−^ → *OOH + OH^−^     ∆G1 (1)

R2: *OOH + e^−^ → *O + OH^−^          ∆G2 (2)

R3: *O + H_2_O + e^−^ → *OH + OH^−^       ∆G3 (3)

R4: *OH + e^−^ →* + OH^−^              ∆G4 (4)

The OER is the reversible process of the ORR, and the reaction process is described as:

R1’: * + OH^−^ → *OH + e^−^           ∆G1’ (5)

R2’: *OH + OH^−^ → *O + H_2_O +e^−^        ∆G2’ (6)

R3’: *O + OH^−^ → *OOH + e^−^            ∆G3’ (7)

R4’: *OOH + OH^−^ → * + O_2_ + H_2_O + e^−^       ∆G4’ (8)

where * denotes the adsorption site of the catalyst, and *OOH, *O, and *OH are the adsorbed intermediates. The free energy change of the ORR (∆Gx:x = 1–4) and of OER (∆Gx’:x = 1–4) can be described by Equations (1)–(8) and calculated based on computational hydrogen electrode (CHE) [40] according to the following equation:

∆G = ∆E + ∆ZPE − T∆S + ∆G_U_ + ∆G_pH,_ (9)

where ∆E, ∆ZPE, and ∆S are the changes in total energy, the zero point energy, and the entropy calculated by DFT. ∆G_U_ is the free energy change due to the applied electrode potential U, it was calculated according to the formula ∆G_U_ = −*ne*U, where *e*, *n*, and U are the transferred electron, the number of *e*, and the applied potential, respectively. ∆G_pH_ is the free energy change due to a change of H^+^ concentration, it was obtained according to the formula ∆G_pH_ = 2.303 K_B_ T × pH (pH = 14 and T = 298.15 K).

The potential-determination step (PDS) in the ORR and OER is defined as the reaction step with the maximum the free energy change [41]. The ORR overpotential (η^ORR^) and OER overpotential (η^OER^) are obtained by Equations (10) and (11):

η^ORR^ = max (ΔG1, ΔG2, ΔG3, ΔG4)/e + 0.401, (10)

The OER theoretical overpotential (η^OER^) is defined as:

η^OER^ = max (ΔG1’, ΔG2’, ΔG3’, ΔG4’)/e − 0.401, (11)

where 0.401 V is the theoretical equilibrium potential when pH = 14 calculated based on the computational hydrogen electrode (CHE) model [40]. For ORR, O_2_(g) + 2H_2_O(l) + 4e^−^ → 4OH^−^(aq), the free energy change ΔG can be calculated by the equation ΔG = 4G_OH_^−^ − [2G_H2O_ + G_O2_]. For OER, 4OH^−^(aq) → O_2_(g) + 2H_2_O(l) + 4e^−^, the free energy change ΔG’ can be calculated by the equation ΔG’ = [2G_H2O_(l) + G_O2_] – 4G_OH_^−^. The free energy of H_2_O(l) is derived as G_H2O (l)_ = G_H2O(g)_ + RT × ln(p/p^0^) since only G_H2O_(g) can be directly obtained by DFT calculations, where R is the ideal gas constant, T = 298.15 K, *p* = 0.035 bar, and p^0^ = 1 bar. The free energy of O_2_(g) has been estimated as G_O2_ = 2G_H2O(l)_ – 2G_H2_ + 4.92 eV. The free energy of OH^−^ was derived as G_OH−_ = G_H2O(l)_ – G_H+_, G_OH_^−^ = G_H2O(l)_ – ½G_H2_ – 2.303 K_B_T × pH. When pH = 14, ΔG = −1.605 eV, ΔG’ = 1.605 eV, the theoretical equilibrium potential is 0.401 V.

**Figure 2:**
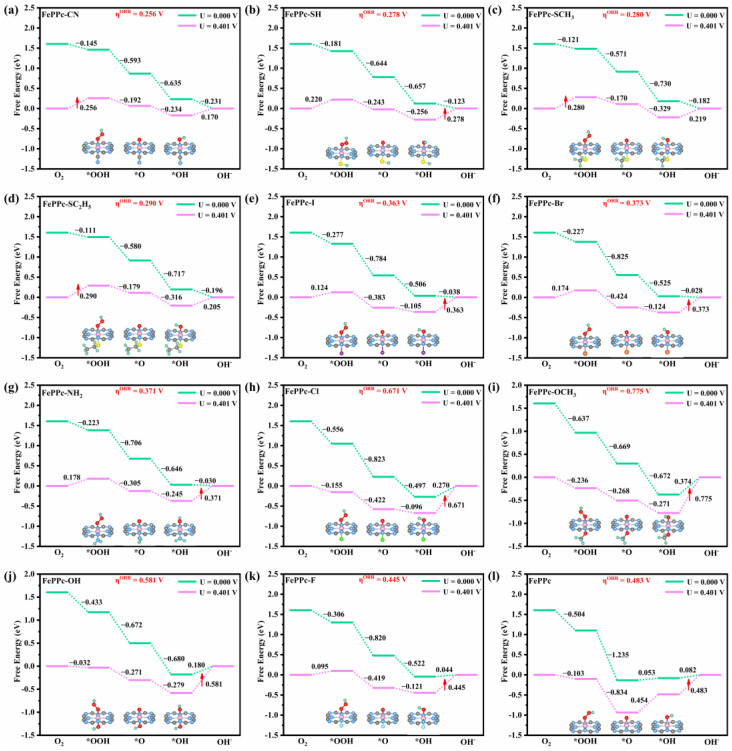
Free energy diagrams of ORR on FePPc and FePPc-L at U = 0 V and U = 0.401 V in alkaline solution. The potential-determination step (PDS) of the ORR is marked with a red upward arrow. The stable geometrical configurations of the intermediates (*OOH, *O, *OH) are also exhibited. The green, gray, blue, red, cyan, yellow, bright green, orange, purple, and pink present H, C, N, O, F, S, Cl, Br, I, and Fe atoms, respectively. (**a**–**k**) Free energy diagrams of ORR on FePPc-L (L = -CN, -SH, -SCH_3_, -SC_2_H_5_, -I, -Br, -NH_2_, -Cl, -OCH_3_, -OH, and -F). (**l**) Free energy diagram of ORR on FePPc.

**Figure 3:**
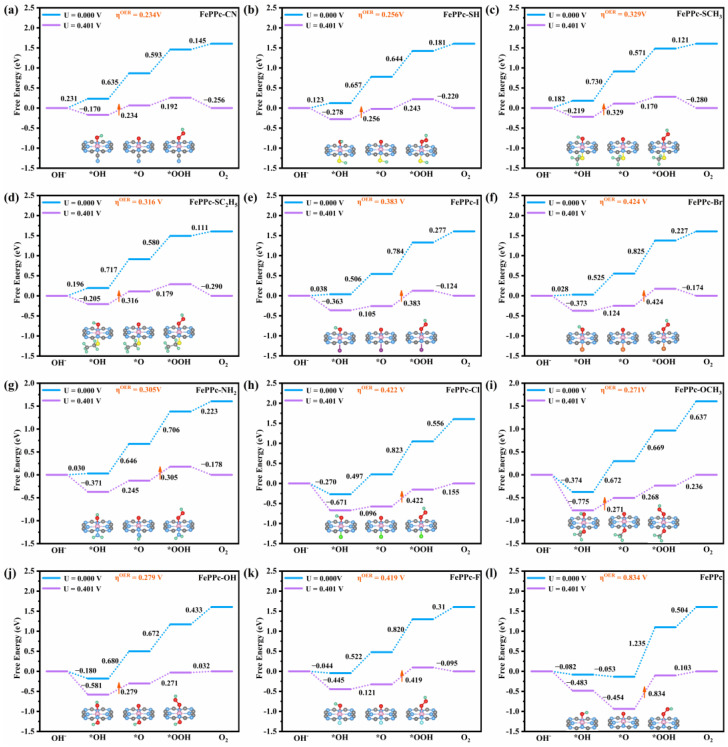
Free energy diagrams of OER on FePPc and FePPc-L at U = 0 V and U = 0.401 V in alkaline solution. The potential-determination step (PDS) of the OER is marked with an orange upward arrow. The stable geometrical configurations of the intermediates (*OOH, *O, *OH) are also exhibited. The green, gray, blue, red, cyan, yellow, bright green, orange, purple, and pink present H, C, N, O, F, S, Cl, Br, I, and Fe atoms, respectively. (**a**–**k**) Free energy diagrams of OER on FePPc-L (L = -CN, -SH, -SCH_3_, -SC_2_H_5_, -I, -Br, -NH_2_, -Cl, -OCH_3_, -OH, and -F). (**l**) Free energy diagram of OER on FePPc.

The Implicit solvation calculations were performed adopting a continuum solvation model of vaspsol [42] to consider the OER/ORR catalytic activity and electronic structure analysis of FePPc and FePPc-L. The free energy diagrams of ORR and OER of FePPc and FePPc-L are drawn as shown in Figure 2 and Figure 3, respectively.

Primarily, we studied the ORR/OER catalytic activity of the FePPc. As shown in Figure 2l, firstly, O_2_ adsorbed on the Fe atom of the FePPc accepted a proton from H_2_O and an electron from the solvent to form *OOH with the change of free energy (∆G1) of −0.504 eV (R1: * + O_2_ + H_2_O + e^−^ → *OOH + OH^−^, ∆G1 = −0.504 eV). Secondly, the *OOH continued to accept an electron and removed an OH^−^ to form *O with ∆G2 of −1.235 eV (R2: *OOH + e^−^ → *O + OH^−^, ∆G2 = −1.235 eV). Subsequently, the *O continued to accept a proton and an electron to form *OH with ∆G3 of 0.053 eV (R3: *O + H_2_O + e^−^ → *OH + OH^−^, ∆G3 = 0.053 eV). Finally, the *OH continued to accept an electron and desorb in the form of OH^−^ from FePPc with ∆G4 of 0.082 eV (R4: *OH + e^−^ →* + OH^−^, ∆G4 = 0.082 eV). R1’ and R2 are exothermic process, and R3 and R4 are endothermic process. It can be obtained that R4 is the PDS, and the η^ORR^ is 0.483 V. The η^ORR^ of FePPc is almost equal to that of Pt(111) (η^ORR^ = 0.50 V) [40]. OER is an inverse process to ORR. As shown in Figure 3l, the whole reaction process is as follows: R1’: * + OH^−^ → *OH + e^−^ (∆G1’ = −0.082 eV), R2’: *OH + OH^−^ → *O + H_2_O + e^−^ (∆G2’ = −0.053 eV), R3’: *O + OH^−^ → *OOH + e^−^ (∆G3’ = 1.235 eV), and R4’: *OOH + OH^−^ → * + O_2_ + H_2_O + e^−^ (∆G4’ = 0.504 eV). It can be obtained that the PDS is R3’and the η^OER^ is 0.834 V. Distinctly, the η^OER^ of FePPc is much higher than that of RuO_2_ (0.42 V) [43]. So FePPc is a good ORR catalyst, but not a good OER catalyst.

As shown in Figure 2a–k, the ORR process of FePPc-L is similar to FePPc. All intermediates are adsorbed on the Fe atom of the catalyst from the side opposite to the axial ligands. For FePPc-L (L = -CN, -SCH_3_, and -SC_2_H_5_), the PDS is R1 (* + O_2_ + H_2_O + e^−^ → *OOH + OH^−^), and for FePPc-L (L = -SH, -I, -Br, -NH_2_, -Cl, -OCH3, -OH, and -F), the PDS is R4 (*OH + e^−^ → * + OH^−^). The η^ORR^ of FePPc-L (L = -CN, -SH, -SCH_3_, -SC_2_H_5_, -I, -Br, -NH_2_, -Cl, -OCH_3_, -OH, and -F) are 0.256 V, 0.278 V, 0.280 V, 0.290 V, 0.363 V, 0.373 V, 0.371 V, 0.671 V, 0.775 V, 0.581 V, and 0.445 V, respectively. Obviously, except for FePPc-L (L = -Cl, -OCH_3_, and –OH), the η^ORR^ of the other FePPc-L (L = -CN, -SC_2_H_5_, -SCH_3_, –SH, -I, -Br, -NH_2_, and -F) are lower than that of the FePPc (0.483 V), especially FePPc-L (L = -CN, -SH, -SCH_3_, and -SC_2_H_5_).

As shown in Figure 3a–k, the OER process of FePPc-L is also similar to FePPc. The PDS is R2’ (*OH + OH^−^ → *O + H_2_O + e^−^) for FePPc-L (L = -CN, -SH, -SCH_3_, -SC_2_H_5_, -OH, and -OCH_3_), the PDS is R3’ (*O + OH^−^ → *OOH + e^−^) for FePPc-L (L = -I, -Br, -NH_2_, -Cl, and -F). The η^OER^ of FePPc-L (L = -CN, -SH, -SCH_3_, -SC_2_H_5_, -I, -Br, -NH_2_, -Cl, -OCH_3_, -OH, and -F) are 0.234 V, 0.256 V, 0.329 V, 0.316 V, 0.383 V, 0.424 V, 0.305 V, 0.422 V, 0.271 V, 0.279 V, and 0.419 V, the η^OER^ of all FePPc-L are much lower than that (0.834 V) of the FePPc, especially FePPc-L (L = -CN, -SH, -SCH_3_, -SC_2_H_5_, -OCH_3_, and -OH). The sum of ORR and OER overpotential (η^ORR + OER^ = η^ORR +^ η^OER^) was used to characterize the bifunctional electrocatalytic activity [44]. The η^ORR + OER^ of FePPc-L (L = -CN, -SH, -SCH_3_, -SC_2_H_5_, -I, -Br, -NH_2_, -Cl, -OCH_3_, -OH, and -F) are 0.490 V: 0.256/0.234 V, 0.534 V: 0.278/0.256 V, 0.609 V: 0.280/0.329 V, 0.606 V: 0.290/0.316 V, 0.746:0.363/0.383 V, 0.797 V: 0.373/0.424 V, 0.676 V: 0.371/0.305 V, 1.093 V: 0.671/0.422 V, 1.046 V: 0.775/0.271 V, 0.860 V: 0.581/0.279 V, 0.864 V: 0.445/0.419 V, which are far lower than that of FePPc (1.317 V: 0.483/0.834 V), IrO_2_ (1.77 V: 0.65/1.12 V), Pt (1.68 V: 1.25/0.43 V) [45]. So FePPc-L (L = -CN, -SH, -SCH_3_, -SC_2_H_5_, -I, -Br, -NH_2_, -Cl, -OCH_3_, -OH, and -F) are promising OER/ORR bifunctional catalysts, especially FePPc-L (L = -CN, -SH, -SCH_3_, and -SC_2_H_5_).

### 2.3. Adsorption Properties and OER/ORR Activity Descriptor

Adsorption-free energy can reflect the adsorption strength of different catalysts to the same substance. For good electrocatalysts, the adsorption strength of the reaction intermediates is moderate, which is conducive to the activation and desorption of the reaction intermediates [41]. In ORR and OER, the adsorption-free energies of *OOH, *O, or *OH (ΔG_*OOH_, ΔG_*O_, or ΔG_*OH_) usually are used as the catalytic activity descriptor of the catalyst [46,47]. ΔG_*OOH_, ΔG_*O_, and ΔG_*OH_ of the FePPc and FePPc-L were calculated by the following equations:

(12) $${\Delta G}_{*\mathrm{OOH}}{=G}_{*\mathrm{OOH}}{-\;G}_{*}{-\;(2G}_{H_{2}O}-\;3(\frac{1}{2}G_{H_{2}}-\;2{.303k}_{B}T\;\times \mathrm{pH})),$$

(13) $${\Delta G}_{*O}{=G}_{*O}{-\;G}_{*}{-\;(G}_{H_{2}O}-\;2(\frac{1}{2}G_{H_{2}}-\;2{.303k}_{B}T\;\times \mathrm{pH})),$$

(14) $${\Delta G}_{*\mathrm{OH}}{=G}_{*O}{-\;G}_{*}{-\;(G}_{H_{2}O}-\;(\frac{1}{2}G_{H_{2}}-\;2{.303k}_{B}T\times \mathrm{pH})),$$

where G_*_, G_*OOH_, G_*O_, and G_*OH_ are the free energies of catalyst, adsorbed intermediates (*OOH, *O and *OH) in alkaline solution. $G_{H_{2}}$ and $G_{H_{2}O}$ are the free energies of H_2_ and H_2_O(l). k_B_ is boltzmann constant, T = 298.15 K and pH = 14. All were obtained according to the CHE model [40]. The adsorption free energy intermediates (ΔG_*OOH_, ΔG_*O_, and ΔG_*OH_, eV) calculated are shown in Table 1.

**Figure 4:**
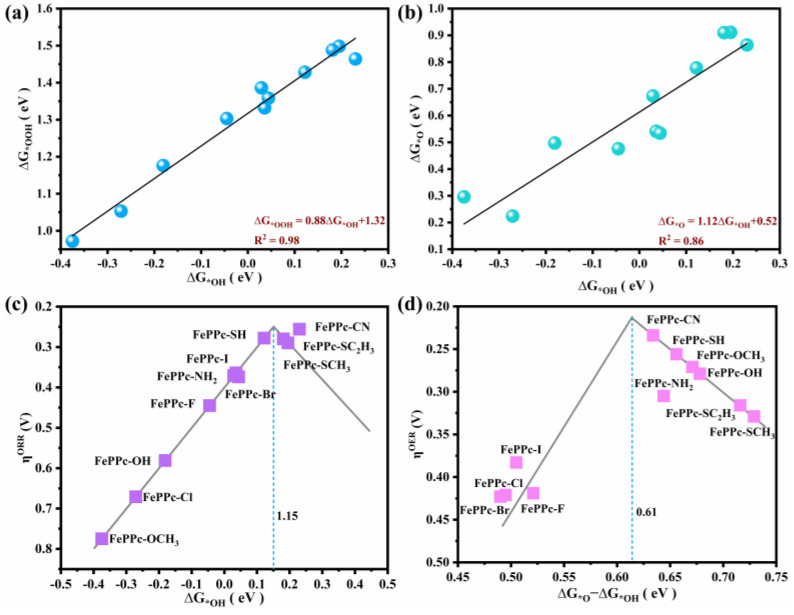
(**a**) The linear relationship of ΔG_*OOH_ vs. ΔG_*OH_. (**b**) The linear relationship of ΔG_*O_ vs. ΔG_*OH_. (**c**) The relationship curve of η^ORR^ and ΔG_*OH_. The blue vertical dotted line represents the most moderate ΔG_*OH_ corresponding to the optimal ORR overpotential. (**d**) The relationship curve of η^OER^ and ΔG_*O_ − ΔG_*OH_. The blue vertical dotted line represents the most moderate ΔG_*O_ − ΔG_*OH_ corresponding to the optimal OER overpotential.

In order to find the OER/ORR bifunctional catalytic activity descriptor of FePPc-L, the linear relationships between ΔG_*OOH_ and ΔG_*OH_, ΔG_*O_, and ΔG_*OH_ were calculated, and they were described as: ΔG_*OOH_ = 0.88ΔG_*OH_ + 1.32 (R^2^ = 0.98), and ΔG_*O_ = 1.12ΔG_*OH_ + 0.52 (R^2^ = 0.86), as shown in Figure 4a,b. It means that there is a good linear relationship both between ΔG_*OOH_ and ΔG_*OH_, ΔG_*O_ and ΔG_*OH_. The volcanic curve relationship between the adsorption free energies of the intermediates and overpotential can describe the ORR and OER catalytic activity of the catalyst [44,48]. In ORR, PDS of most of FePPc-L (L = -SH, -I, -Br, -NH_2_, -Cl, -OCH_3_, -OH, and -F) is R4 (*OH + e^−^ → * + OH^−^). In OER, the PDS of half of FePPc-L (L = -CN, -SH, -SCH_3_, -SC_2_H_5_, -OCH_3_, and -OH) are R2’(*OH + OH^−^ → *O + H_2_O + e^−^), the PDS of other FePPc-L(L = -I, -Br, -NH_2_, -Cl, and -F) is R3’(*O + OH^−^ → *OOH + e^−^). So, we mapped the volcanic curve of η^ORR^ vs. ΔG_*OH_, and η^OER^ vs. (ΔG_*O_ − ΔG_*OH_) as shown in Figure 4c and Figure 4d, respectively. From Figure 4c, it can be found that the data does not follow a typical volcano curve, but a plateau. On the left branch of the curve, η^ORR^ decreases with the decrease of adsorption strength of FePPc-L to intermediate *OH, the PDS is R4 (*OH + e^−^ → * + OH^−^). On the right branch of the curve, η^ORR^ has no clear change trend with the increase of adsorption strength of FePPc-L to intermediate *OH, which is limited by the number of catalysts. The PDS is R1 (* + O_2_ + H_2_O + e^−^ → *OOH + OH^−^). When ΔG_*OH_ = 0.15 eV, this corresponds to the optimal ORR overpotentials of 0.25 V. FePPc-CN (η^ORR^ = 0.256 V), FePPc-SH (η^ORR^ = 0.278 V), FePPc-SCH_3_ (η^ORR^ = 0.280 V), and FePPc-SC_2_H_5_ (η^ORR^ = 0.278 V) are near the top of the volcanic curve and show better ORR catalytic performance. From Figure 4d, it can be seen that there is a similar volcano curve relationship between η^OER^ and (ΔG_*O_ − ΔG_*OH_). On the left side of the curve, η^OER^ decreases with the increase G_*O_ − ΔG_*OH_, these FePPc-L (L = -Br, -Cl, -I, and -F) have stronger adsorption for intermediate *O, and the PDS of FePPc-L is R3’ (*O + OH^−^ → *OOH + e^−^). On the right branch of the curve, η^OER^ increases with the increase G_*O_ − ΔG_*OH_, FePPc-L (L = -CN, -SH, -SCH_3_, -SC_2_H_5_, -OCH_3_, and -OH) have weaker adsorption for intermediate *O, and the PDS is R2’ (*OH + OH^−^ → *O + H_2_O + e^−^). When ΔG_*O_ − ΔG_*OH_ = 0.61 eV, this corresponds to the optimal OER overpotentials of 0.22 V. FePPc-CN (η^OER^ = 0.234 V), FePPc-SH (η^OER^ = 0.255 V), FePPc-OCH_3_ (η^OER^ = 0.271 V), and FePPc-OH (η^OER^ = 0.279 V) are near the top of the volcanic curve and show excellent OER catalytic performance. Obviously, among all FePPc-L, FePPc-CN, FePPc-SH, FePPc-SCH_3_, and FePPc-SC_2_H_5_ exhibit excellent ORR/OER bifunctional catalytic activity.

### 2.4. Electronic Properties Analysis

To deeply explore the origin of the OER/ORR bifunctional catalytic activity, we analyzed the electronic structure of FePPc-L, especially for FePPc-CN, FePPc-SH, FePPc-SCH_3_, and FePPc-SC_2_H_5_.

Firstly, we analyzed the Bader charge of the Fe atoms (Q_Fe_), the total Bader charge of four N atoms connected to Fe atom in FePPc and FePPc-L, and the total Bader charge of axial ligands (Q_L_) in FePPc-L as shown in Table 2. It is found that the Fe atoms in FePPc-L showed smaller positive Bader charge in most of FePPc-L than that in FePPc (1.345|e|), especially for FePPc-CN, FePPc-SH, FePPc-SCH_3_, and FePPc-SC_2_H_5_. All Q_L_ of FePPc-L are negative, which indicates that the axial ligands exhibit an electron-withdrawing inductive effect. It is following the fact that the electronegativity of Fe (1.83) is smaller than that of coordinating atoms in axial ligands of FePPc-L: C (2.550) < S (2.580) < I (2.660) < Br (2.960) < N (3.040) < Cl (3.160) < O (3.440) < F (3.980) [49]. Furthermore, we analyzed the spin magnetic moment of the Fe atom (M_Fe_) in FePPc and FePPc-L. It is found that the M_Fe_ changes greatly in FePPc-L compared with that in FePPc (1.866 μ_B_). For FePPc-CN, FePPc-SH, FePPc-SCH_3_, and FePPc-SC_2_H_5_, their smaller electronegativity of coordinating atoms in axial ligands corresponds to smaller M_Fe_. Therefore, the electronegativity of coordinating atoms in axial ligands has a great influence on the charge and magnetic moment of Fe atoms, which may affect the OER/ORR catalytic activity.

**Figure 5:**
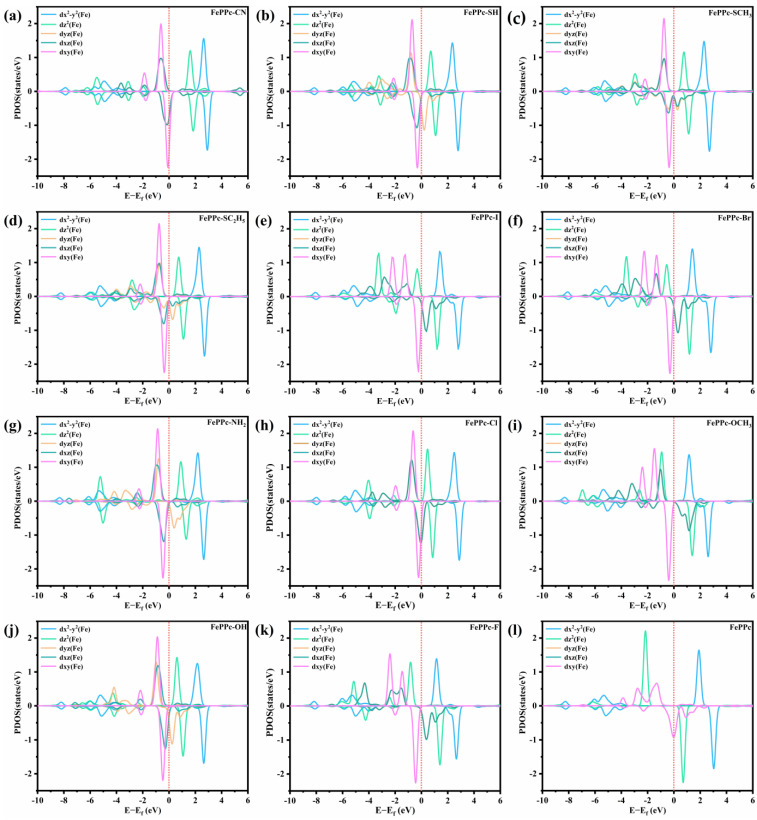
(**a**–**k**) PDOS analysis of FePPc-L (L = -CN, -SH, -SCH_3_, -SC_2_H_5_, -I, -Br, -NH_2_, -Cl, -OCH_3_, -OH, and -F). (**l**) PDOS analysis of FePPc.

**Figure 6:**
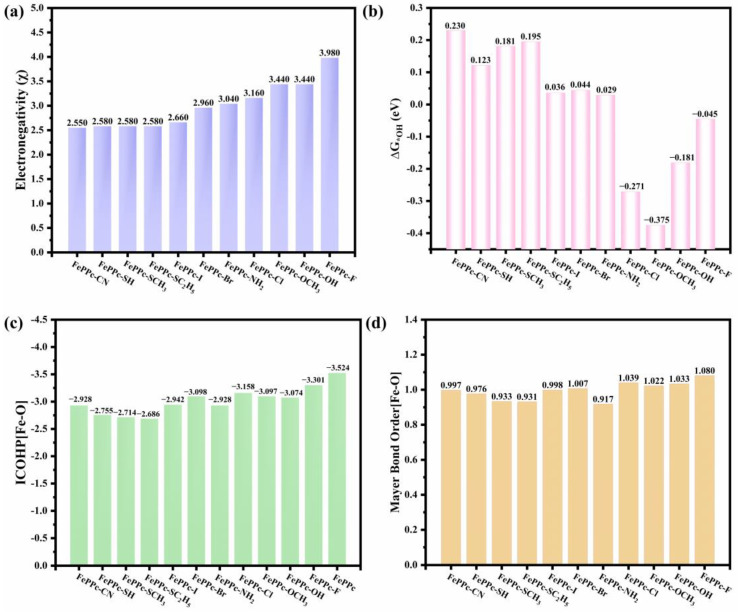
(**a**) Electronegativity of coordinating atoms in axial ligands of various FePPc-L. (**b**) ΔG_*OH_ of FePPc-L. (**c**) Integrated crystal orbital Hamilton population (ICOHP) of Fe-O bond in *OH of FePPc-L. (**d**) Mayer bond order of Fe-O bond in *OH of FePPc-L.

We also analyzed the projected density of states (PDOS) of the Fe 3d orbitals (dx^2^-y^2^, dz^2^, dxz, dyz, and dxy) in FePPc and FePPc-L as shown in Figure 5. Compared with FePPc, for FePPc-L (L = -I, -Br, -OCH_3_, and -F), the polarization of the 3d orbitals is enhanced, which is consistent with bigger electronegativity of coordinating atoms in axial ligands, and the bigger magnetic moment of Fe atom (M_Fe_). Polarization of the 3d orbitals is weaker for other FePPc-L (L = -CN, -SH, -SCH_3_, and -SC_2_H_5_), which is consistent with smaller electronegativity of coordinating atoms in axial ligands and the smaller M_Fe_ as shown in Figure 6a and Table 2. For FePPc-L, the dx^2^-y^2^, dz^2^, dyz, and dxz orbitals all split and became narrower. dz^2^, dyz, and dxz orbitals shift to a higher energy level relative to the fermi level (E_f_) in different degrees. The distribution Polarization of dz^2^, dyz, and dxz orbitals near E_f_ also decreases, especially for FePPc-CN, FePPc-SH, FePPc-SCH_3_, and FePPc-SC_2_H_5_. The ORR/OER intermediates OOH, O, and OH are adsorbed on the iron atom of FePPc-L from the side opposite to the axial ligand through the interaction between the P orbit of oxygen atom and the dz^2^, dyz and dxz orbits of iron atom. It is well known that the dz^2^, dyz and dxz orbitals of Fe atom extend in the axial direction in FePPc-L. The Axial ligands are bonded to Fe atoms by interaction with dz^2^, dyz, and dxz orbitals of the Fe atom, which affects the distribution of the dz^2^, dyz and dxz orbitals of the Fe atom. Axial ligands with different electronegative coordination atoms have different effects on dz^2^, dyz, and dxz orbitals of the Fe atom. The axial ligands with a smaller electronegativity of the coordination atom (-CN, -SH, -SCH_3_, and -SC_2_H_5_) reduce the distribution of dz^2^, dyz, and dxz orbitals near E_f_, thereby weakening the adsorption strength of the catalyst to the intermediate and enhancing the catalytic activity of catalysts. This means that the dz^2^, dyz, and dxz orbitals play a major role in OER/ORR catalytic activity [50]. Therefore, dz^2^, dyz, and dxz orbitals can be used as a descriptor of the catalytic activity of FePPc-L.

**Figure 7:**
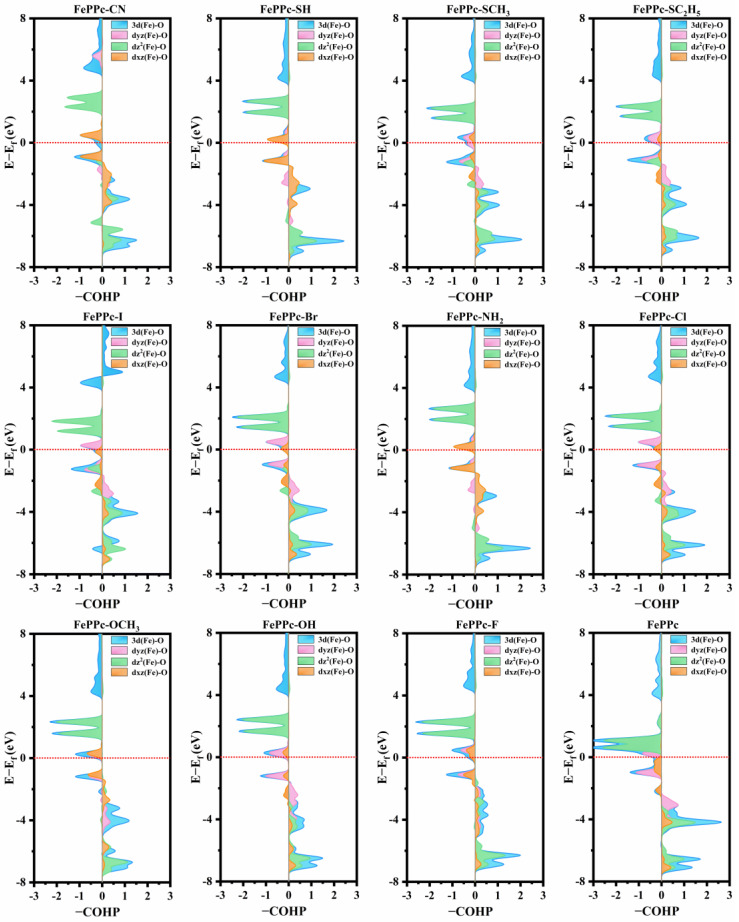
Crystal orbital Hamilton population (COHP) of the Fe-O bond in *OH of FePPc and FePPc-L. The horizontal red dotted line indicates the E_f_. Positive contribution to the right represents the bonding states, while negative contribution to the left represents the anti-bonding states.

In addition, the increasing trend of the electronegativity of coordinating atoms in axial ligands of FePPc-L is roughly consistent with the decreasing trend of the ΔG_*OH_ of FePPc-L as shown in Figure 6a,b. For FePPc-CN, FePPc-SH, FePPc-SCH_3_, and FePPc-SC_2_H_5_, their small electronegativity of coordinating atoms in axial ligands corresponds to their small adsorption strength for *OH. We further analyzed the bond strength of Fe-O bond in *OH of FePPc and FePPc-L based on crystal orbital Hamiltonian (COHP) [51] as shown in Figure 7. It is found that the bonding states distribution below E_f_ of Fe-O, dyz (Fe)-O, dz^2^ (Fe)-O, and dxz (Fe)-O in *OH of FePPc-L are less than that in FePPc, the anti-bonding states below E_f_ of Fe-O bond, dyz (Fe)-O, dz^2^ (Fe)-O, and dxz (Fe)-O in *OH are more than that in FePPc, especially for FePPc-CN, FePPc-SH, FePPc-SCH_3_, and FePPc-SC_2_H_5_. It is also illustrated by the integral COHP of the Fe-O [ICOHP (Fe-O)] in *OH of FePPc and FePPc-L as shown in Figure 6c. The increasing trend of the electronegativity of coordinating atoms in axial ligands of FePPc-L is roughly consistent with the increasing trend of the ICOHP (Fe-O) of *OH in FePPc-L as shown in Figure 6a,c. For FePPc-CN, FePPc-SH, FePPc-SCH_3_, and FePPc-SC_2_H_5_, the small electronegativity of coordinating atoms in axial ligands corresponds to the weak adsorption strength of *OH. It once again shows that the dz^2^, dyz, and dxz orbitals play a major role in OER/ORR catalytic activity. The electronegativity of the coordination atom in axial ligands can affect the distribution of the dz^2^, dyz, and dxz orbitals of the Fe atom, thereby weakening the adsorption strength of the catalyst to the intermediate and enhancing OER/ORR catalytic activity of catalysts.

What needs to be noted is that the ΔG_*OH_ of FePPc-Cl and FePPc-OCH_3_ is smaller than that of other FePPc-L, which means the adsorption strength for *OH is stronger. However, their ICOHP(Fe-O) of *OH are not very big, which is probably because of their stronger solvation effect. As shown in Figure S2a–c, the ΔG_*OOH_, ΔG_*OH_, and ΔG_*OH_ of FePPc and FePPc-L in aqueous solution all are smaller than that in vacuum, particularly for FePPc-Cl and FePPc-OCH_3_, all ΔG_*OOH_, ΔG_*O_, and ΔG_*OH_ descend too much. This may be the reason why the η^ORR^ of FePPc-Cl and FePPc-OCH_3_ are higher in aqueous solution as in Figure S2d.

Mayer bond order is a very effective chemical bond analysis method [52,53]. The Mayer bond order of the Fe-O bond of the *OH of all FePPc-L were also calculated. The growth trend of the Mayer bond order of the Fe-O bond and the electronegativity of coordinating atoms in axial ligands of FePPc-L is broadly consistent as shown in Figure 6a,d. It means that the small the electronegativity of coordinating atoms in axial ligands of FePPc-L corresponds the weak Fe-O bond of *OH, and a small adsorption strength for *OH. For FePPc-CN, FePPc-SH, FePPc-SCH_3_, and FePPc-SC_2_H_5_, the small Mayer bond order of the Fe-O bond of the *OH corresponds to the small electronegativity of coordinating atoms in axial ligands.

## 3. Computational Methods

Spin-polarized density functional theory calculations are performed using the Vienna ab initio simulation package (VASP5.4.4) [54,55] combined with the projector augmented wave (PAW) method [56]. The electron-correction interactions were described by the generalized gradient approximation (GGA) of Perdew-Burke-Ernzerhof (GGA-PBE) [57]. The van der Waals (vdW) interactions were treated via Grimme’s DFT-D3 correction method [58]. The plane wave cutoff energy was 500 eV. In the process of geometric optimization, the convergence criterion of force is 0.01 eVÅ^−1^, energy is 10^−5^ eV, and the vibrational frequency calculations are 10^−7^ eV. A vacuum layer is set as 20 Å to prevent the interaction between adjacent layers. The k-point mesh based on the Monkhorst-Pack scheme [59] was set to 5 × 5 × 1 and 10 × 10 × 1 to optimize the structure and calculate the electronic structure, respectively. The implicit solvation calculations were performed adopting a continuum solvation model of vaspsol [42]. The interaction between two atoms is analyzed by the Crystal orbital Hamilton population (COHP) [51] and the Mayer bond order [52,53].

## 4. Conclusions

In summary, through performing DFT calculations, we studied the ORR/OER bifunctional catalytic activity of iron polyphthalocyanine with a series of axial ligands (L = -CN, -SH, -SCH_3_, -SC_2_H_5_, -I, -Br, -NH_2_, -Cl, -OCH_3_, -OH, and -F) with different electronegative coordination atoms on the Fe atom (FePPc-L) in alkaline media. It is expected to understand how the electronegativity of coordination atoms in axial ligands affacts the catalytic activity of the FePPc-L and design effective ORR/OER bifunctional catalysts. The important results are as follows:

Compared with other FePPc-L, FePPc-CN, FePPc-SH, FePPc-SCH_3_, and FePPc-SC_2_H_5_ exhibit more excellent ORR/OER bifunctional catalytic activities. Their ORR/OER overpotential are 0.256 V/0.234 V, 0.278 V/0.256 V, 0.280 V/0.329 V, and 0.290 V/0.316 V, respectively, which are much lower than that of the FePPc (0.483 V/0.834 V). The analysis of the electronic structure of the above catalysts shows that the electronegativity of the coordination atoms in the axial ligand is small, resulting in less distribution of dz_2_, dyz, and dxz orbitals near E_f_, weak orbital polarization, small charge and magnetic moment of the central Fe atom, and weak adsorption strength for *OH. All these prove that the axial ligands with appropriate electronegative coordination atoms can affect the electronic structure of FeN_4_ in FePPc, adjust the adsorption of catalyst to intermediates, and modify the ORR/OER bifunctional catalytic activities. The conclusions are generally in good agreement with our study goal. So this is an effective strategy of designing ORR/OER bifunctional electrocatalysts with low cost and high efficiency.

## Figures and Tables

**Table 1:** Adsorption free energy intermediates (ΔG_*OOH_, ΔG_*O_, and ΔG_*OH_, eV) in alkaline solution.

Structure	ΔG_*OOH_	ΔG_*O_	ΔG_*OH_
FePPc-CN	1.464	0.864	0.230
FePPc-SH	1.428	0.778	0.122
FePPc-SCH_3_	1.488	0.910	0.181
FePPc-SC_2_H_5_	1.498	0.911	0.195
FePPc-I	1.332	0.541	0.036
FePPc-Br	1.358	0.534	0.044
FePPc-NH_2_	1.386	0.673	0.029
FePPc-Cl	1.053	0.224	−0.271
FePPc-OCH_3_	0.972	0.296	−0.375
FePPc-OH	1.176	0.497	−0.181
FePPc-F	1.303	0.476	−0.045
FePPc	1.105	−0.137	−0.083

**Table 2:** Q_Fe_, Q_N_, and Q_L_ represent the Bader charge of the Fe atom, the total Bader charge of four N atoms connected to Fe atom, and the total Bader charge of axial ligands in FePPc-L. The positive and negative bader charges present the charge accumulation and depletion, respectively. M_Fe_ represents the magnetic moment of Fe atoms in FePPc and FePPc-L.

Structure	Q_Fe_(|e|)	Q_N_(|e|)	Q_L_(|e|)	M_Fe_(μ_B_)
FePPc-CN	1.223	−4.515	−0.537	0.532
FePPc-SH	1.179	−4.420	−0.203	0.779
FePPc-SCH_3_	1.130	−4.399	−0.089	0.753
FePPc-SC_2_H_5_	1.079	−4.368	−0.039	0.756
FePPc-I	1.228	−4.578	−0.436	2.267
FePPc-Br	1.235	−4.546	−0.541	2.323
FePPc-NH_2_	1.248	−4.442	−0.128	0.789
FePPc-Cl	1.250	−4.516	−0.445	0.833
FePPc-OCH_3_	1.340	−4.537	−0.403	2.303
FePPc-OH	1.362	−4.462	−0.391	0.833
FePPc-F	1.417	−4.544	−0.702	2.695
FePPc	1.345	−4.748	--	1.866

## Data Availability

Data are contained within the article.

## Supplementary Materials

The following supporting information can be downloaded at: https://www.mdpi.com/article/10.3390/molecules29010210/s1, Figure S1: The top and side view of the unit cell of FePPc. The green, gray, blue, and pink present H, C, N, and Fe atoms, respectively. Figure S2: (a–c) ΔG_*OOH_, ΔG_*O_, and ΔG_*OH_ of FePPc and FePPc-L in vacuum and in aqueous solution. (d–f) η^ORR^, η^OER^, and η^ORR + ηOER^ of FePPc and FePPc-L in vacuum and in aqueous solution. Table S1: The lattice parameters (in Å) of cell of FePPc and FePPc-L. Average bond length of Fe-N bond (d_Fe-N_, in Å) of FePPc and FePPc-L.
