# Supplementary material for: Theoretical Study on ORR/OER Bifunctional Catalytic Activity of Axial Functionalized Iron Polyphthalocyanine
Source: Molecules. 2023 Dec 30;29(1):210. doi: 10.3390/molecules29010210 (PMC10780174; doi:10.3390/molecules29010210)
Supplement: Supplementary file 1 [file molecules-29-00210-s001.zip › molecules-2758878-supplementary.pdf]

Supporting Information

Theoretical study on ORR/OER bifunctional catalytic activity of axial functionalized iron polyphthalocyanine

Guilin Wang [1,2], Xiaoqin Feng [1], Rongrong Ren [1], Yuxin Wang [1], Jie Meng [1], and Jianfeng Jia [1,*]

[1] Key Laboratory of Magnetic Molecules and Magnetic Information Materials (Ministry of Education), School of Chemistry and Material Science, Shanxi Normal University, Taiyuan 030031, China.

[2] Department of Physics and Electronic Engineering, Yuncheng University, Yuncheng 044000, China

* Correspondence: jiajf@dns.sxnu.edu.cn

Tel: +86 357 2052468

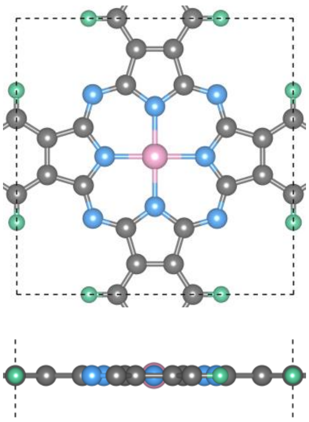

Figure S1. The top and side view of the unit cell of FePPc. The green, gray, blue, and pink present H, C, N, and Fe atoms, respectively.

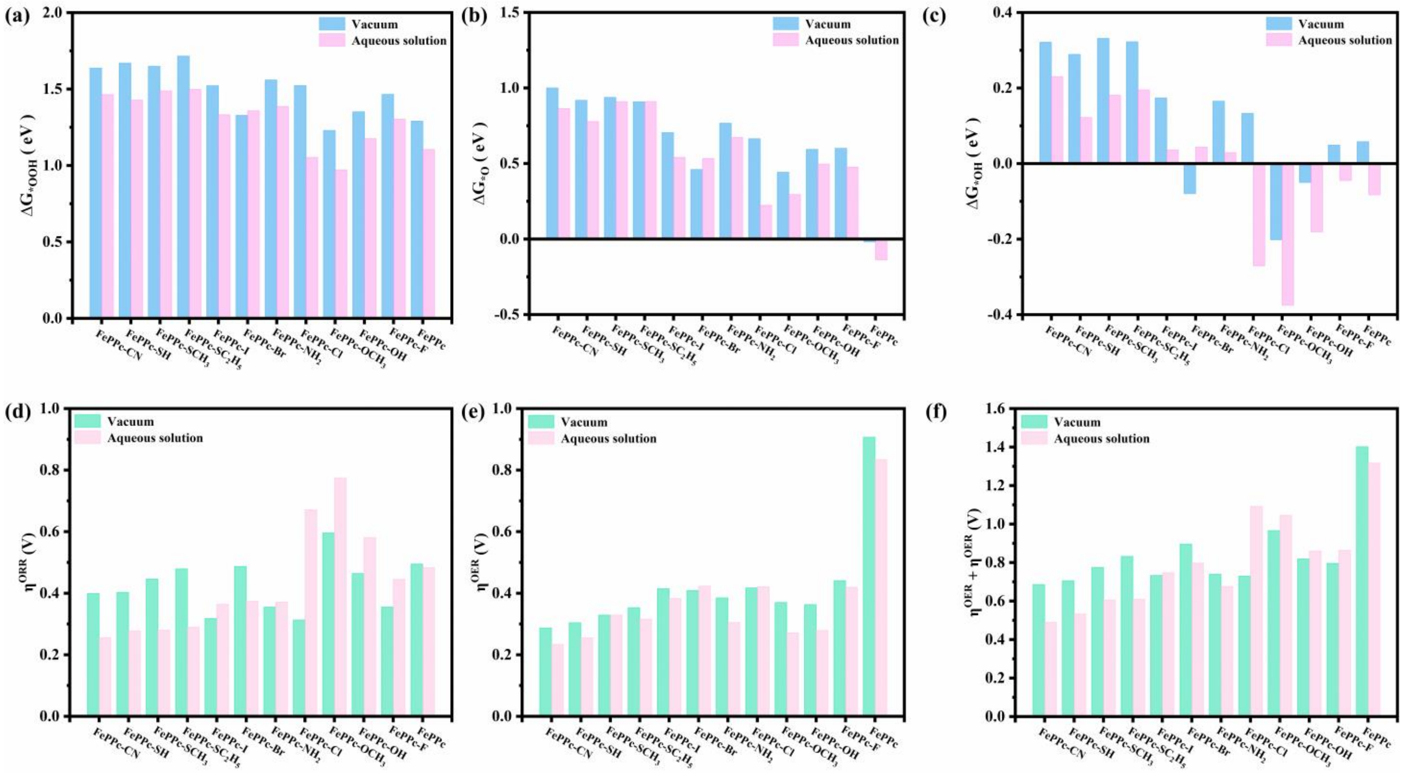

Figure S2 (a)–(c) ΔG_{*OOH}, ΔG_{*O}, and ΔG_{*OH} of FePPc and FePPc-L in vacuum and in aqueous solution. (d)–(f) η^{ORR}, η^{OER}, and η^{ORR} + η^{OER} of FePPc and FePPc-L in vacuum and in aqueous solution.

Table S1. The lattice parameters (in Å) of cell of FePPc and FePPc-L. Average bond length of Fe–N bond (**d_{Fe-N}**, in Å) of FePPc and FePPc-L.

Structure	Lattice parameter	Lattice parameter	d_{Fe-N}
FePPc-CN	a=10.64	b=10.64	1.93
FePPc-SC_2H_5	a=10.65	b=10.65	1.95
FePPc-SCH_3	a=10.65	b=10.66	1.94
FePPc-SH	a=10.64	b=10.66	1.94
FePPc-I	a=10.65	b=10.65	1.96
FePPc-Br	a=10.64	b=10.64	1.93
FePPc-NH_2	a=10.64	b=10.66	1.94
FePPc-Cl	a=10.65	b=10.65	1.96
FePPc-OCH_3	a=10.66	b=10.66	1.97
FePPc-OH	a=10.64	b=10.67	1.94
FePPc-F	a=10.66	b=10.66	1.96
FePPc	a=10.66	b=10.66	1.93
